# Cognitive performance in idiopathic intracranial hypertension and relevance of intracranial pressure

**DOI:** 10.1093/braincomms/fcab202

**Published:** 2021-09-02

**Authors:** Olivia Grech, Andrew Clouter, James L Mitchell, Zerin Alimajstorovic, Ryan S Ottridge, Andreas Yiangou, Marianne Roque, Abd A Tahrani, Matthew Nicholls, Angela E Taylor, Fozia Shaheen, Wiebke Arlt, Gareth G Lavery, Kimron Shapiro, Susan P Mollan, Alexandra J Sinclair

**Affiliations:** Metabolic Neurology, Institute of Metabolism and Systems Research, University of Birmingham, Edgbaston B15 2TT, UK; Centre for Endocrinology, Diabetes and Metabolism, Birmingham Health Partners, Birmingham B15 2TH, UK; Department of Psychology, Nottingham Trent University, Nottingham NG1 5LT, UK; Metabolic Neurology, Institute of Metabolism and Systems Research, University of Birmingham, Edgbaston B15 2TT, UK; Centre for Endocrinology, Diabetes and Metabolism, Birmingham Health Partners, Birmingham B15 2TH, UK; Department of Neurology, University Hospitals Birmingham NHS Foundation Trust, Birmingham B15 2TH, UK; Metabolic Neurology, Institute of Metabolism and Systems Research, University of Birmingham, Edgbaston B15 2TT, UK; Centre for Endocrinology, Diabetes and Metabolism, Birmingham Health Partners, Birmingham B15 2TH, UK; Birmingham Clinical Trials Unit, College of Medical and Dental Sciences, University of Birmingham, Birmingham B15 2TT, UK; Metabolic Neurology, Institute of Metabolism and Systems Research, University of Birmingham, Edgbaston B15 2TT, UK; Department of Neurology, University Hospitals Birmingham NHS Foundation Trust, Birmingham B15 2TH, UK; Birmingham Neuro-Ophthalmology Unit, University Hospitals Birmingham NHS Foundation Trust, Birmingham B15 2TH, UK; Centre for Endocrinology, Diabetes and Metabolism, Birmingham Health Partners, Birmingham B15 2TH, UK; Institute of Metabolism and Systems Research, University of Birmingham, Birmingham B15 2TT, UK; Department of Endocrinology and Diabetes, University Hospitals Birmingham NHS Foundation Trust, Birmingham B15 2TH, UK; Centre for Endocrinology, Diabetes and Metabolism, Birmingham Health Partners, Birmingham B15 2TH, UK; Institute of Metabolism and Systems Research, University of Birmingham, Birmingham B15 2TT, UK; Centre for Endocrinology, Diabetes and Metabolism, Birmingham Health Partners, Birmingham B15 2TH, UK; Institute of Metabolism and Systems Research, University of Birmingham, Birmingham B15 2TT, UK; Centre for Endocrinology, Diabetes and Metabolism, Birmingham Health Partners, Birmingham B15 2TH, UK; Institute of Metabolism and Systems Research, University of Birmingham, Birmingham B15 2TT, UK; Centre for Endocrinology, Diabetes and Metabolism, Birmingham Health Partners, Birmingham B15 2TH, UK; Institute of Metabolism and Systems Research, University of Birmingham, Birmingham B15 2TT, UK; National Institute for Health Research (NIHR), Birmingham Biomedical Research Centre, University of Birmingham and University Hospitals Birmingham NHS Foundation Trust, Birmingham B15 3GW, UK; Centre for Endocrinology, Diabetes and Metabolism, Birmingham Health Partners, Birmingham B15 2TH, UK; Institute of Metabolism and Systems Research, University of Birmingham, Birmingham B15 2TT, UK; Centre for Human Brain Health, School of Psychology, University of Birmingham, Birmingham B15 2TT, UK; Birmingham Neuro-Ophthalmology Unit, University Hospitals Birmingham NHS Foundation Trust, Birmingham B15 2TH, UK; Metabolic Neurology, Institute of Metabolism and Systems Research, University of Birmingham, Edgbaston B15 2TT, UK; Centre for Endocrinology, Diabetes and Metabolism, Birmingham Health Partners, Birmingham B15 2TH, UK; Department of Neurology, University Hospitals Birmingham NHS Foundation Trust, Birmingham B15 2TH, UK

**Keywords:** idiopathic intracranial hypertension, cognition, intracranial pressure, headache, visual field

## Abstract

Cognitive impairments have been reported in idiopathic intracranial hypertension; however, evidence supporting these deficits is scarce and contributing factors have not been defined. Using a case-control prospective study, we identified multiple domains of deficiency in a cohort of 66 female adult idiopathic intracranial hypertension patients. We identified significantly impaired attention networks (executive function) and sustained attention compared to a body mass index and age matched control group of 25 healthy female participants. We aimed to investigate how cognitive function changed over time and demonstrated that deficits were not permanent. Participants exhibited improvement in several domains including executive function, sustained attention and verbal short-term memory over 12-month follow-up. Improved cognition over time was associated with reduction in intracranial pressure but not body weight. We then evaluated cognition before and after a lumbar puncture with acute reduction in intracranial pressure and noted significant improvement in sustained attention to response task performance. The impact of comorbidities (headache, depression, adiposity and obstructive sleep apnoea) was also explored. We observed that body mass index and the obesity associated cytokine interleukin-6 (serum and cerebrospinal fluid) were not associated with cognitive performance. Headache severity during cognitive testing, co-morbid depression and markers of obstructive sleep apnoea were adversely associated with cognitive performance. Dysregulation of the cortisol generating enzyme 11β hydroxysteroid dehydrogenase type 1 has been observed in idiopathic intracranial hypertension. Elevated cortisol has been associated with impaired cognition. Here, we utilized liquid chromatography-tandem mass spectrometry for multi-steroid profiling in serum and cerebrospinal fluid in idiopathic intracranial hypertension patients. We noted that reduction in the serum cortisol:cortisone ratio in those undergoing bariatric surgery at 12 months was associated with improving verbal working memory. The clinical relevance of cognitive deficits was noted in their significant association with impaired reliability to perform visual field tests, the cornerstone of monitoring vision in idiopathic intracranial hypertension. Our findings propose that cognitive impairment should be accepted as a clinical manifestation of idiopathic intracranial hypertension and impairs the ability to perform visual field testing reliably. Importantly, cognitive deficits can improve over time and with reduction of intracranial pressure. Treating comorbid depression, obstructive sleep apnoea and headache could improve cognitive performance in idiopathic intracranial hypertension.

## Teaser

Grech et al. report cognitive impairments in idiopathic intracranial hypertension compared to body mass index matched controls. Deficits improved following lumbar puncture and 12 months weight loss intervention, and were associated with headache severity, depression, obstructive sleep apnoea and cortisol. Performance of visual field testing was impacted by impaired attention.

## Graphical Abstract

**Graphical Abstract:**
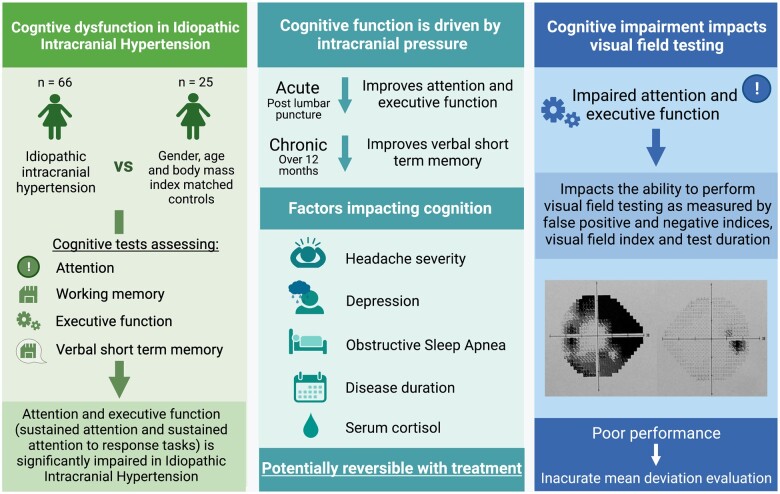

## Introduction

Idiopathic intracranial hypertension is an increasingly prevalent, disabling disease that is being recognized as associated with multiple co-morbidities.^1–3^ The condition is characterized by papilloedema resulting from raised intracranial pressure, without an evident structural lesion, with the potential for permanent visual loss.^4^^,^^5^ Debilitating headache is a dominant feature in IIH.^6^ Latest insights into pathophysiology suggest that IIH is a neurometabolic disease, with a defined unique signature of androgen excess and increased activity of the cortisol generating enzyme 11β hydroxysteroid dehydrogenase type 1 (11β HSD 1).^7–11^

From as early as 1986, disturbances of cognitive performance were formally noted as part of the IIH clinical phenotype.^12^ IIH patients commonly reported cognitive symptoms including problems with thinking or memory.^13^ Cognitive function, however, is not commonly recognized or addressed during the routine evaluation of those with IIH,^2^^,^^14^ and the clinical tool, the mini mental state examination, is insensitive in this population.^13^ Several small cohort studies have formally assessed memory and cognition in IIH.^13^^,^^15–19^ Studies have shown deficits in key areas such as memory, learning, visuospatial skills, concentration, language and executive function.^13^^,^^15–19^ A retrospective review of 10 cases found impairment in long-term memory, delayed recall and retention.^13^ Others have demonstrated deficits in reaction time and processing speed.^15^ Moreover, they found that deficits persisted at 3 months.^20^ The factors contributing to cognitive dysfunction in IIH and the reversibility in the longer term have not been determined. There are a number of potential factors that have been shown to influence cognitive function, which we suggest are likely to be relevant in IIH. These include obesity^21^^,^^22^ and the resulting pro-inflammatory state,^23^ headache,^24^^,^^25^ depression,^26^ sleep apnoea^27^ and hormonal dysregulation.^28^ Importantly, the role of intracranial pressure in cognitive performance is not established.

To address this gap in the knowledge, we aimed to conduct a prospective case-control study, documenting the extent of cognitive deficits in multiple domains including attention, executive function, short-term and working memory in adults with active IIH compared to matched controls. To explore previous findings noting deficits are irreversible in IIH,^20^ we aimed to investigate how cognitive function changed over 12 months. We also sought to identify if weight management interventions, a treatment that can achieve sustained remission of IIH, impacted cognitive function.^29^ Using lumbar puncture as an intervention to reduce intracranial pressure temporarily, we aimed to investigate the acute effect on cognition, in both active IIH and healthy controls. Additionally, we sought to explore the relationship between potentially confounding comorbidities (headache, depression, weight, obstructive sleep apnoea) and the steroid metabolic profile, with cognitive function in IIH. Finally, we aimed to evaluate the impact of cognitive dysfunction on performing visual field testing, the cornerstone of IIH visual monitoring.

## Materials and methods

The study design was a case-controlled study comparing cognitive domains between control and IIH participants at baseline. IIH participants were prospectively evaluated at 12 months as part of a sub-study of the IIH: WT trial, a controlled, randomized, parallel-group, multi-centre trial comparing the effects of a bariatric surgery pathway versus a community weight management intervention.^30^ IIH participants were identified from neurology and ophthalmology clinics from seven UK National Health Service hospitals and controls recruited through advertising on social media. The protocol has been published elsewhere.^30^ The study was approved by the National Research Ethics Committee [West Midlands–The Black Country approved IIH: WT (14/WM/0011)]. The trial was registered with ISRCTN (ISRCTN40152829). All participants provided written informed consent. A subgroup of IIH participants were enrolled into an intervention study to evaluate the acute effect of lumbar puncture on cognition.

### Participants

Women with IIH (18–55 years), with a body mass index [BMI = weight (kg)/height (m)^2^] ≥35 kg/m^2^ were eligible if they had a clinical diagnosis of active IIH (papilloedema Frisen grade >1 and lumbar puncture opening pressure >25 cmCSF on the date of baseline visit following a formal diagnosis of IIH).^2^ Meeting the diagnostic criteria for IIH,^31^ included intracranial pressure ≥25 cmCSF, papilloedema and normal brain imaging including magnetic resonance venography or computed tomography venography (apart from radiological signs of raised intracranial pressure) at recruitment. Detailed inclusion and exclusion criteria have been published (Supplementary material).^30^ Participants with a clinical meaningful central visual field loss were excluded as determined by the ocular reading centre as this may have impacted the ability to perform the cognitive testing. Controls included women with obesity (BMI ≥35 kg/m^2^) aged between 18 and 55 years with analogous exclusion criteria to the IIH participants.

### Clinical measurements

#### Study visit

At baseline medical history, BMI and a headache diary to record monthly headache days and severity [numerical rating scale 0–10, with 10 denoting the maximum pain] were documented. Headache severity (numerical rating scale) at the time of cognitive testing was also documented. The visual assessments included visual perimetry [Humphrey 24–2 Swedish Interactive Thresholding Algorithm central automated perimetry] and performance reliability markers (false positive and negative, visual field index, test duration and mean deviation). Papilloedema was quantified through optic nerve head imaging using optical coherence tomography (Spectralis^TM^, Heidelberg Engineering, Germany) to evaluate the total average peripapillary retinal nerve fibre layer. Participants completed questionnaires to assess health-related quality of life [measured by Rand short-form (SF)-36, the hospital anxiety and depression scores (HAD-A and -D), and headache disability (HIT-6)]. Cognitive testing was then performed using a battery of cognitive tests (Supplementary Fig. 1). On the same day, a lumbar puncture was performed in the left lateral decubitus position to record the lumbar puncture opening pressure and to collect CSF (10 ml) with matched serum. IIH participants were randomized to one of the two trial arms (community weight management intervention or a bariatric surgery pathway). For a sub-cohort of control and IIH participants, the sustained attention to response task was repeated (within 30 min) of lumbar puncture.

#### Cognitive testing

Detailed evaluation of cognitive function was conducted using a bespoke battery of cognitive tests. These included the attention network test^32^ (modified to examine interactions^33^^,^^34^). The attention network test measures three domains of attention: alerting, orienting and executive function (specifically selective attention and the ability to ignore conflicting stimuli). Verbal short-term memory was measured using a word span task, and the operation span task was used to measure verbal working memory.^35^^,^^36^ The sustained attention task and sustained attention to response task were used to measure sustained attention and executive function (specifically the ability to override the prepotent motor response).^37^^,^^38^ Raven’s Standard Progressive Matrices was used to evaluate intelligence. Tests were delivered by a trained member of the research team in the clinical research facility under standard lighting conditions (Supplementary materials, Supplementary Fig. 1).

#### Obstructive sleep apnoea testing

Obstructive sleep apnoea is associated with obesity and IIH^39^; therefore, we conducted 12 h of overnight recording at home on two nights, using a multichannel cardiorespiratory sleep apnoea device (ResMed ApneaLink Air, UK). The data were scored by a sleep specialist and the session with the longest recording time was selected and quality controlled by a second specialist in sleep medicine. Sleep studies were scored in accordance with the American Academy of Sleep Medicine guidelines.^40^ The apnoea–hypopnoea index, lowest desaturation, time spent with oxygen saturation <90% and overnight desaturation index were recorded.

#### Pro-inflammatory cytokine profile analysis

Interleukin (IL)-6, a pro-inflammatory cytokine associated with obesity,^41^ was analysed in serum and CSF. Following collection, samples were centrifuged within 30 min (10 min 1500g for blood, 800g for CSF) at 4°C and stored at –80°C. All samples underwent a single freeze–thaw cycle. Interleukin 6 was quantified by ELISA as per manufacturer's instructions using the Human interleukin 6 DuoSet ELISA (R&D Systems, Cat No. DY206, UK). The kit is a solid-phase sandwich ELISA, and well optical density was determined using the Wallac microplate reader and Wallac 420 workstation software set to a 450 nm wavelength.

#### Steroid measurements

Serum and CSF steroid concentrations were analysed by multi-steroid profiling using liquid chromatography-tandem mass spectrometry, as previously described.^7^^,^^42^^,^^43^ These measurements included cortisol and cortisone.

### Statistical analysis

Statistical analysis of the cognitive tests was performed using R (version 4.0.3, Vienna, Austria) and the ‘ez’ package. Binomial correct/error data were transformed as a logistic regression (i.e. the log of the odds ratio [percentage correct/(1 – percentage correct)]) for the purposes of logistic regressions or the computation of correlations, so as to not violate the assumptions of standard statistical tests especially near the floor and ceiling. Statistical analyses were carried out using linear models or factorial ANOVA for within- or between-groups designs, as appropriate. Comparisons for the study were limited to those pre-planned. Results reported reflect where multiple cognitive tests yielded a significant result (*P* < 0.05) in the same cognitive domain. Isolated significant results are documented in the supplementary tables.

Statistical analysis of clinical parameters was undertaken on Prism [Prism 8 for MacOS, Graphpad, LCC, Version 8.4.0 (455)]. Baseline clinical characteristics were compared between control and IIH participants using a two-tailed unpaired *t*-test. For comparisons between baseline and follow, two-tailed paired *t* tests were used, and unpaired *t* tests were used at follow-up between trial arms. Statistical analysis of the correlations of cognitive and clinical parameters was undertaken on SPSS [Armonk, NY: IBM Corp. Version 25.0 (2017)]. Clinical measurements were correlated with cognitive performance at baseline and against absolute changes using two-tailed Spearman’s rank order correlation. Statistical significance was considered at *P *<* *0.05 level (two-tailed) in which *P *<* *0.05 = *, *P *<* *0.01 = **, *P *<* *0.001 = ***. Missing data were excluded from the analysis. In the prospective analysis, only those with matched data at baseline and 12 months were included. Likewise, only those participants who had both tests performed pre- and post-lumbar puncture were analysed.

### Data availability statement

Data will be available beginning 12 months and ending 3 years after publication of this article to researchers whose proposed use of the data is approved by the original study investigators.

## Results

### Cognitive impairment in idiopathic intracranial hypertension participants relative to controls

Initially, a detailed evaluation of cognitive domains in IIH compared to control participants with obesity was conducted. Twenty-five controls and 66 IIH participants were included in the study (Table 1). All participants were female, the mean (SD) age of the controls was 39.0 (9.3) years and 32.0 (7.8) years for the IIH participants. The controls were BMI-matched to IIH participants, with the mean (SD) BMI of 44.0 (5.3) in controls and 43.9 (7.0) in IIH. Medication use was recorded (Supplementary Table 1). Both IIH and controls had comparable intelligence (Raven’s Standard Progressive Matrices; Table 1).

**Table 1:** Demographic and clinical characteristics of study participants

	Control	IIH
Demographic characteristics	Mean (SD), *n*	Mean (SD), *n*
Age ^***^	39.0 (9.3), 25	32.0 (7.8), 66
Body mass index (kg/m^2^)	44.0 (5.3), 25	43.9 (7.1), 66
Opening lumbar puncture pressure (cmCSF) ^***^	23.0 (4.4), 24	34.7 (5.7), 66
Closing lumbar puncture pressure (cmCSF) ^***^	16.2 (3.0), 22	19.4 (3.8), 61
Headache severity day of test ^***^	0.5 (1.4), 25	3.5 (2.8), 61
HAD anxiety score	9.0 (5.2), 25	10.3 (4.9), 65
HAD depression score	6.9 (4.9), 25	7.6 (4.5), 65
Physical summary of measures score (SF-36) ^**^	39.2 (14.5), 19	28.7 (12.7), 60
Mental summary of measures score (SF-36)	40.8 (13.1), 19	37.7 (11.0), 60
Apnoea–hypopnea index	14.9 (9.1), 17	14.1 (20.5), 40
Overnight desaturation index	15.2 (9.5), 17	14.1 (19.7), 40
Average unit alcohol/week	1.5 (3.2),19	2.8 (4.3), 41
Average exercise per week (h:min)	05:12 (0.3), 19	03:49 (0.2), 41
Smoker, yes (no)	2 (16), 18	15 (26), 41
Raven’s correct (total %)	39.9 (9.1), 11	41.5 (10.2), 33
Raven’s time (minutes)	18.4 (3.9), 11	20.9 (7.8), 33
Employment		
% Employment	16 (60%)	35 (53%)
<£10 000	4 (27%)	5 (14%)
£10 001 to £30 000	6 (40%)	16 (46%)
>£30 001	5 (31%)	11 (31%)
Not disclosed	1 (7%)	3 (9%)

Quantitative data are expressed as mean (SD), *n* and compared with unpaired *t*-test. Categorical data are expressed as *n* (%) and compared with chi-squared test or Fisher’s exact test. No significant difference found between groups except where indicated.

* = *P *<* *0.05,

** = *P *<* *0.01,

*** = *P *<* *0.001.

IIH participants demonstrated cognitive impairment in multiple domains compared to controls [attention network test (alerting effect *P *=* *0.023 flanker effect *P *=* *0.007), sustained attention (reaction time *P *=* *0.003) and sustained attention to response tasks (target correct *P *=* *0.031); Table 2].

**Table 2:** Within- and between-groups comparisons for cognitive test performance between IIH and controls

Cognitive test	Measure	Control		IIH		Difference (Controls–IIH)	*P* (between groups)
		Mean score (SD), *n*	*P* (within)	Mean score (SD), *n*	*P* (within)		
Attention network alerting (yes–no)	RT	–18 (22), 19	<0.001	–17 (24), 53	<0.001	–1	0.861
	Correct	0.008 (0.028), 19	0.017	–0.001 (0.028), 53	0.845	0.009	0.023
Attention network orienting (valid–invalid)	RT	–69 (47), 19	<0.001	–83 (35), 53	<0.001	14	0.186
	Correct	0.004 (0.025), 19	0.313	0.012 (0.030), 53	<0.001	–0.008	0.602
Attention network flankers (incongruent–congruent)	RT	125 (45), 19	<0.001	126 (46), 53	<0.001	–1	0.940
	Correct	–0.036 (0.064), 19	<0.001	–0.048 (0.094), 53	<0.001	0.013	0.007
Sustained attention	RT	434 (44), 22	–	476 (53), 37	–	–42	0.003
	Correct	0.997 (0.004), 22	–	0.996 (0.005), 37	–	0.002	0.149
	Target correct	0.999 (0.002), 22	–	0.997 (0.005), 37	–	0.002	0.069
Sustained attention to response	RT	358 (57), 21	–	395 (82), 43	–	–38	0.063
	Correct	0.920 (0.077), 21	–	0.892 (0.071), 43	–	0.028	0.057
	Target correct	0.772 (0.0254), 21	–	0.615 (0.289), 43	–	0.157	0.031
Word span	Correct	0.631 (0.121), 24	–	0.625 (0.146), 52	–	0.005	0.868
Operation span	Correct	0.611 (0.180), 25	–	0.565 (0.183), 51	–	0.046	0.248

Scores are expressed as mean (SD) and compared with within (paired) or between (unpaired) analysis of variance, repeated-measures analysis of variance, *t*-tests or *z*-tests, as appropriate.

‘–’ indicates that within-group comparison is not applicable.

Correct = proportion correct; mean *P* (within) = *P*-value for within-groups difference for the attention network test conditions validates expected condition effects; *P* (between) = *P*-value for between-groups difference; RT = reaction time (milliseconds); Target correct = proportion correct on target-present trials, errors of omission/commission.

As expected, both the participant and the control groups showed the standard attention network test effects (the altering effect, the orientating effect and the flanker effect). We did, however, identify significant differences in the ability to make correct responses, between IIH and controls for the alerting effect (analysis of variance *P = *0.0234). Here, the IIH participants did not show improved performance as a result of the alerting tone. In addition, we noted a significant difference in the flanker effect between IIH and controls (*P *=* *0.007), with IIH participants being less able to maintain their attention and more prone to distraction by the peripheral stimuli (Table 2, Supplementary Table 2).

**Figure 1:**
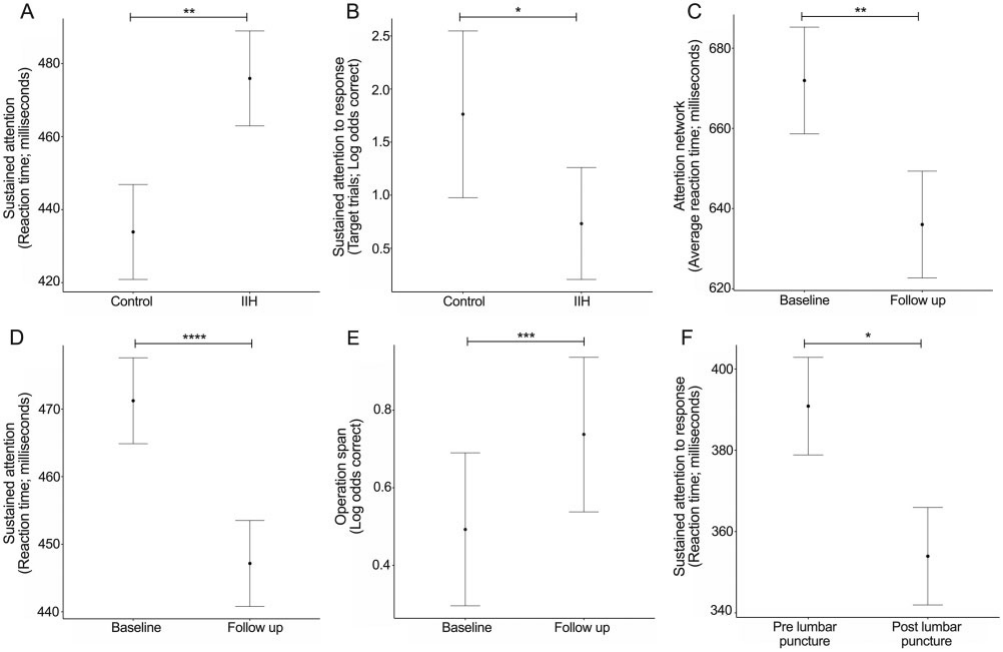
**Cognitive task performance differences between control and IIH participants, baseline and follow-up and pre- and post-lumbar puncture**. (**A**) Sustained attention reaction time is higher in IIH than controls at baseline (434 ms control versus 47s6 ms IIH; *P* = 0.003). (**B**) IIH participants made more errors of commission than controls at baseline (proportion correct 0.772 control versus 0.615 IIH; *P* = 0.031). (**C**) Reaction times during attention network tests are significantly lower at follow-up than at baseline (baseline 695 ms versus 12 months 657 ms, *P* = 0.005). (**D**) Sustained attention reaction times are also reduced at follow-up compared to baseline (baseline 471 ms versus 12 months 447 ms, *P* < 0.001). (**E**) Performance in operation span task is improved at follow-up compared to baseline (baseline 0.588 versus 0.650 follow-up, *P* = <0.001). (**F**) Sustained attention to response task reaction time is lower post-lumbar puncture than pre-lumbar puncture (baseline 391 ms versus 12 months 354 ms, *P* = 0.004). Scores expressed as mean (SD) and compared with within/paired or between/unpaired analysis of variance, repeated-measures analysis of variance, *t*-tests, or *z*-tests, as appropriate. * = *P* <0.05, ** = *P* <0.01, *** = *P* <0.001.

Performances in the sustained attention and sustained attention to response tasks were significantly different between controls and IIH (*P *=* *0.003 and *P *=* *0.031, respectively; Fig. 1A and B and Table 2). We noted a pattern of slower reaction times and increased errors indicating impairments in sustaining attention, timely evaluation of a stimuli and making (as well as inhibiting) a response. No significant impairments were identified between control and IIH participants for verbal short-term and working memory.

### Recovery of cognitive performance over time in idiopathic intracranial hypertension participants

Previously, cognitive dysfunction in IIH was noted to be a fixed deficit reported over a 3-month evaluation period.^20^ We sought to prospectively re-evaluate IIH participants after 12 months. Over the 12-month follow-up, we noted a mean (SD) reduction in BMI of 4.68 (5.92) kg/m^2^ with a mean (SD) reduction in intracranial pressure of 6.00 (8.45) cmCSF.

Overall, performance improved in multiple tests of cognitive function (speed of responses, stimulus evaluation, action selection and inhibition, sustained attention and working memory; Table 3). Marked improvements were noted in the attention network test for each individual task condition after 12 months [average attention network mean (SD) reaction time at baseline 695 (83) ms versus 12 months 657 (83) ms, *P *=* *0.005; Fig. 1C, Table 3]. Similarly, improvements in reaction times were demonstrated for the sustained attention task [baseline mean (SD) 471 (52) ms versus 12 months 447 (44) ms, *P *<* *0.001; Fig. 1D]. In addition, verbal working memory, measured by the operation span task significantly improved (mean percentage correct 59% at baseline and 65% at follow-up, *P *<* *0.001; Fig. 1E, Table 3).

**Table 3:** Comparison of cognitive performance at baseline and follow-up in IIH participants

Cognitive test	Measure	*n*	Baseline	Follow-up	Change	*P*
			Score (SD)	Score (SD)		
Attention network test (averaged)	RT	39	695 (83)	657 (91)	–38	0.005
	Correct	39	0.964 (0.062)	0.966 (0.081)	0.002	0.162
Sustained attention	RT	34	471 (52)	447 (44)	–24	< 0.001
	Correct	34	0.996 (0.005)	0.994 (0.008)	–0.001	0.210
	Target correct	34	0.982 (0.025)	0.970 (0.049)	–0.012	0.085
Sustained attention to response	RT	36	391 (73)	354 (59)	–37	0.074
	Correct	36	0.902 (0.066)	0.896 (0.094)	–0.006	0.192
	Target correct	36	0.648 (0.282)	0.656 (0.314)	0.009	0.176
Word span	Correct	42	0.620 (0.152)	0.645 (0.132)	0.024	0.080
Operation span	Correct	39	0.588 (0.193)	0.650 (0.169)	0.062	<0.001

Scores are expressed as mean (SD) and compared with paired *t*-tests or *z*-tests, as appropriate.

Correct = proportion correct; RT = reaction time; Target correct = proportion correct on target-present trials errors of omission/commission.

No meaningful differential improvements were noted in a sub-analysis comparing those assigned to either the community weight management intervention or bariatric surgery pathway (Supplementary Tables 4 and 5). This was noted in the context of differential weight loss and reduction in intracranial pressure been the groups [BMI decreased by mean (SD) –2.2 (8.8) kg/m^2^ versus –23.0 (14.7) kg/m^2^, *P *<* *0.0001 and intracranial pressure fell by mean (SD) –2.2 (4.9) cmCSF versus –9.0 (10.2) cmCSF, *P *=* *0.005 respectively; Supplementary Table 4].

The reduction in intracranial pressure over 12 months was noted to correlate significantly with improved sustained attention to response task performance (errors of omission *r* = –0.361, *P *=* *0.043). There was no correlation with change in BMI. Taken together, these results demonstrate that cognitive deficits in IIH are not permanent and may, in part, be driven by intracranial pressure.

### Sustained attention improves following lumbar puncture

Sustained attention to response task performance is impaired in IIH participants compared to controls (Fig. 1B, Table 2). Therefore, we assessed performance in a sub-cohort (IIH *n* = 43, controls *n* = 22), before and immediately after a lumbar puncture where intracranial pressure is acutely reduced. In the IIH sub-cohort, the lumbar puncture opening pressure was mean (SD) 34.2 (5.1) cmCSF and closing pressure 19.7 (3.9) cmCSF (returned to normal reference range). Reaction times significantly improved following lumbar puncture in IIH participants [pre-lumbar puncture mean (SD) time 391 (3) ms versus post 354 (59) ms, *P *=* *0.004; Fig. 1F]. This occurred without any changes in total errors or errors to the target stimuli (Table 4). Control subjects with normal intracranial pressure did not improve. Importantly, these results suggest that an acute reduction in the CSF pressure in IIH participants leads to an almost immediate improvement in timely evaluation of stimuli and making (as well as inhibiting) a response.

**Table 4:** Sustained attention to response task performance pre- and post-lumbar puncture in IIH participants

Task measure	Pre-lumbar puncture	Post-lumbar puncture	Change	*P*
	Mean score (SD), *n*	Mean score (SD), *n*		
RT	391 (3), 36	354 (59), 36	–0.037	0.004
Correct	0.902 (0.066), 36	0.896 (0.094), 36	–0.006	0.180
Target correct	0.648 (0.282), 36	0.656 (0.314), 36	0.009	0.346

Scores are expressed as mean (SD) and compared with paired *t*-tests or *z*-tests, as appropriate.

Correct = proportion correct; RT = reaction time; Target correct = proportion correct on target-present trials errors of omission/commission.

### Factors contributing to cognitive dysfunction in idiopathic intracranial hypertension

IIH is associated with factors that have been noted in other conditions to influence cognition.^44^ To gain an understanding of other potential contributors to the cognitive dysfunction demonstrated in the IIH cohort, we evaluated the association of contributors with IIH cognitive performance.

#### Headache

We first considered the impact of headache severity at the time of cognitive testing. The attention network test percentage correct was correlated with headache severity (both in the presence of an alerting tone *r* = –0.380, *P *=* *0.005, and in the presence of an incongruent flanker, *r* = –0.341, *P *=* *0.012). Headache diary measurements (reflecting headaches over the previous month) including monthly mean headache severity, frequency and HIT-6 were not associated with cognitive deficits.

#### Obesity

Obesity occurs in over 90% of patients with IIH and has also been implicated in cognitive dysfunction.^45^^,^^46^ Among this IIH cohort, there was no relationship between BMI or the obesity associated pro-inflammatory cytokine interleukin 6^41^ (serum or CSF levels) and cognition.

#### Depression

Reduced quality of life and depression are reported in IIH.^47^^,^^48^ Depression has previously been linked to impaired cognition^26^; therefore, we investigated the association with anxiety and depression scores (HAD-A and -D) and the quality of life (SF-36) mental component summary score and physical component summary score (PCS). The depression score (HAD-D) was associated with the attention network test average reaction time (*r* = 0.3, *P = *0.03). Additionally, the quality of life PCS also correlated with the attention network test average reaction time (*r* = –0.333, *P = *0.021). HAD-D, PCS and mental component summary scores were also correlated with sustained attention to response task total proportion correct (HAD-D; *r* = –0.324, *P *=* *0.036; *r* = 0.402, PCS; *P *=* *0.014 and mental component summary score; *r* = 0.353, *P *=* *0.032). No associations were found with the HAD-A score. Thus, scores reflecting depression and impaired quality of life were associated with impaired performance on the attention network test and the sustained attention to response task.

#### Obstructive sleep apnoea

Obstructive sleep apnoea and overnight hypoxia have previously been demonstrated to impact on cognitive function.^49^ Sixty three per cent of the IIH cohort met the criteria for obstructive sleep apnoea (Supplementary Table 3). Working memory was associated with a higher apnoea–hypopnea index and more profound oxygen overnight desaturations (higher apnoea–hypopnea index; *r* = 0.659, *P *=* *0.001, lowest desaturation overnight; *r* = –0.619, *P *=* *0.002, oxygen desaturation index; *P *=* *0.003 and *r* = 0.637). After 12 months of weight loss intervention, we noted that improvements in obstructive sleep apnoea indices were associated with improved executive function. Specifically, reductions in the apnoea–hypopnea index and overnight oxygen desaturation index were associated with improved sustained attention task reaction times (*r* = 0.618, *P *=* *0.043 and *r* = 0.655, *P *=* *0.029, respectively); no significant associations were found between change in intracranial pressure and BMI and change in apnoea–hypopnea index and overnight oxygen desaturation index.

#### Disease duration

Duration of active IIH (time from confirmed diagnosis to baseline study visit) was associated with executive function. The sustained attention to response task reaction time (*r* = 0.304, *P *=* *0.048) and sustained attention task (*r* = –0.451, *P *=* *0.007) correlated with disease duration. Attention network test performance percentage correct was also associated with disease duration (alerting no *r* = –0.311, *P *=* *0.023, flanker incongruent *r* = –0.304, *P *=* *0.027).

#### Glucocorticoids and cognition in idiopathic intracranial hypertension

Chronic cortisol excess is known to be associated with cognitive impairment.^50^^,^^51^ Serum cortisol levels were noted to correlate with executive function in IIH (verbal working memory *r* = –0.55, *P *=* *0.002). As expected, the serum cortisol : cortisone ratio fell significantly more in those undergoing bariatric surgery compared to those in the community weight management intervention group over 12 months [mean (SD) change –0.5785 (2.039) versus 0.7094 (0.709), respectively *P *=* *0.0223].^9^ This consequent reduction in serum cortisol at 12 months significantly correlated with executive function (verbal working memory *r* = –0.63, *P *=* *0.009). Over the 12-month follow-up, the reduction in BMI was also associated with improved executive function (verbal working memory *r* = –0.374, *P *=* *0.035). Multivariate regression analysis identified that the association of the improvement in verbal working memory with serum cortisol remained significant (*r*^2^ = 0.735, *P *=* *0.011) after adjusting for BMI (*P *=* *0.106) and apnoea–hypopnea index (*P *=* *0.811).

#### Impact of cognitive dysfunction on ability to perform visual field assessments

**Figure 2:**
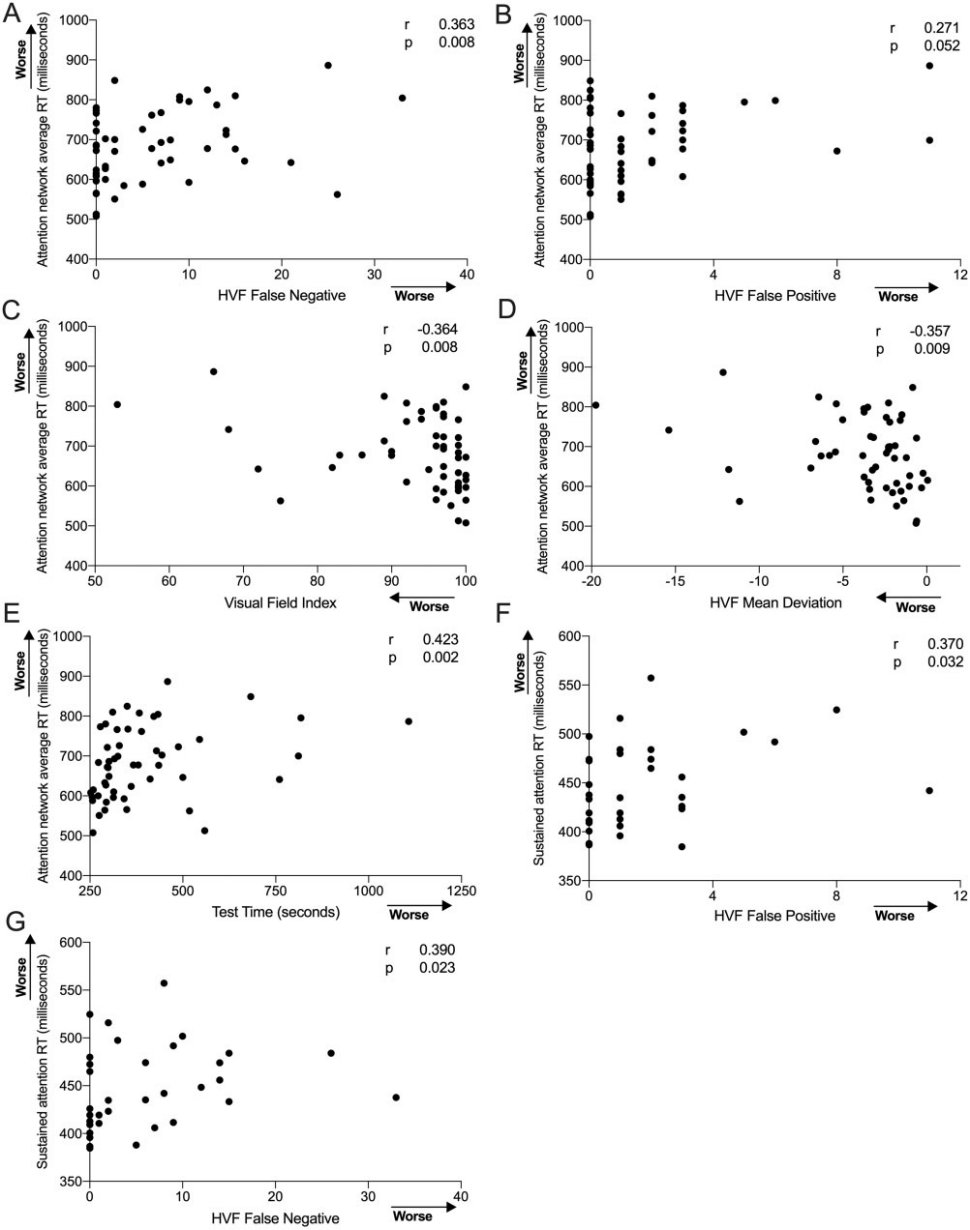
**Correlation between vision measurements, attention network and sustained attention task performance in IIH at baseline. Attention network test and sustained attention to response task times are correlated with HVF measurements in IIH participants**. (**A**) Average attention network test reaction time and HVF false negative. (**B**) Average attention network test reaction time and HVF false positive. (**C**) Average attention network test reaction time and visual field index. (**D**) Average attention network test reaction time and HVF mean deviation. (**E**) Average attention network test reaction time and test time duration. (**F**) Sustained attention to response task reaction time and HVF false positive. (**G**) Sustained attention to response task reaction time and HVF false negative. Non-parametric Spearman’s rank performed to calculate correlation, *r* and *P* values. HVF = Humphrey visual field; RT = reaction time.

IIH patients are known to perform frequently unreliably on visual field tests, traditionally, the principal measure of visual decline in IIH.^52^ We have observed that attention network test reaction times are slower in IIH (Table 2). We hypothesized that this would contribute to the poor reliability in performing visual field tests by IIH patients. Humphrey visual field mean deviation scores, a measure of visual field function, significantly correlated with attention network test reaction time (*r* = –0.357, *P *=* *0.009; Fig. 2D, Table 5). Further we identified that attention network test performance was significantly associated with the ability to perform a visual field test accurately as measured by the false negative indices, visual field index and test duration (false negative *r* = 0.363, *P *=* *0.008, visual field index *r* = –0.364, *P *=* *0.008 and test duration *r* = 0.423, *P *=* *0.002, Fig. 2A, C and E, Table 5). Humphrey visual field measurements also correlated with sustained attention to response task performance (Fig. 2F and G). Headache severity at the time of testing was not significantly associated with any visual field measurements. None of the patients met the criteria for exclusion based on clinical meaningful central visual field loss that would have impaired their ability to perform screen-based cognitive assessments [worse eye mean deviation (SD) –3.6 dB (3.7) and best eye –2.6 dB (4.2)]. No relationship was found between cognition and markers of papilloedema (optical coherence tomography measure of retinal nerve fibre layer thickness); this is surprising as we have noted that intracranial pressure is significantly associated with cognitive performance (and intracranial pressure has been previously associated with extent of papilloedema^53^). This lack of association between cognition and papilloedema in this study may reflect variability in the temporal relationship between changes in papilloedema and intracranial pressure observed in individual patients.

**Table 5:** Association between attention network test reaction times and Humphrey visual field test measurements at baseline

HVF test measurement	Mean (SD), *n*	*P*	*r*
Mean deviation	–3.6 (3.7), 65	0.009	–0.357
False negative	5.4 (7.3), 65	0.008	0.363
False positive	1.6 (2.3), 65	0.052	0.271
Visual field index	93.7 (9.2), 65	0.008	–0.364
Test duration (seconds)	377.3 (158.4), 65	0.002	0.423

Humphrey visual field test measurements at baseline are shown as mean (SD), *n* is association with attention network task average response time calculated via non-parametric Spearman’s rank.

HVF = Humphrey visual field.

## Discussion

We report the most comprehensive evaluation of cognition in IIH to date. Our results reveal reversible multidomain cognitive deficits in IIH. The prominent deficit was significant impairment in sustained attention and executive function in IIH participants as compared to controls. Further, markers of executive function (sustained attention) improved over time in association with falling intracranial pressure. Executive function (sustained attention) also improved with acute reduction in intracranial pressure following lumbar puncture. Comorbidities of headache severity, depression and obstructive sleep apnoea were associated with impaired cognitive performance. Treatment to address these factors is thus likely to improve cognition impairment in IIH. Of key relevance to the evaluation of vision in IIH is the finding that impaired attention reaction times were associated with reduced reliability to perform a visual field assessment.

### Executive function and attention is significantly impaired in idiopathic intracranial hypertension

IIH patients have long described cognitive ‘fogging’. Emerging evidence from six previous smaller studies noted cognitive dysfunction in IIH, but detailed assessments including the relationship to duration of disease and co-morbidities were not described.^13^^,^^15–19^ The prior studies also did not have the benefit of a BMI-matched control group, which could have introduced a confounding effect of obesity.^21^^,^^22^ We have conducted detailed cognitive testing in the largest IIH cohort to date, and for the first time compared to a gender, BMI and intelligence matched control group. IIH participants exhibited impaired executive function and sustained attention with slower reaction times and more errors. We did not identify deficits in working or verbal short-term memory in IIH compared to obesity matched control participants. Our findings using a sensitive, bespoke cognitive battery are supported by the previous smaller studies that used a more standard neuropsychological test battery.^17^^,^^18^^,^^20^ Our results reflect the reported patient experience of difficulty concentrating or maintaining focus.^13^

### Idiopathic intracranial hypertension cognitive deficits diminish over time

Previous studies of cognition in IIH have not agreed as to whether cognitive deficits are permanent, and few provided long-term follow-up data. Yri et al.^20^ showed no resolution of cognitive deficits over 3 months duration. However, the case series by Sorensen et al.^12^ (20 participants) showed improvement in learning and memory following routine medical and surgical treatment of IIH. Importantly, our study showed that performance in the majority of cognitive domains improved over a 12-month follow-up period. Importantly, executive function (sustained attention and sustained attention to response tasks) the principal deficit in IIH compared to controls, improved over 12 months. We did not demonstrate improvement in verbal working memory although our test may have lacked the sensitivity.

Factors contributing to reversibility of cognitive deficits are of interest. Weight loss has been previously documented to improve cognition in obese individuals (in the absence of IIH).^54^ Over the 12-month study period, the IIH participants lost weight and intracranial pressure fell, more so in those randomized to a bariatric surgery pathway compared to those in a community weight management intervention. However, no significant differences in cognitive performance were noted between these two trial arms. The numbers in this sub-analysis were likely too small to draw firm conclusions. The obesity-associated pro-inflammatory cytokine interleukin 6 (in both serum and CSF) was not associated with adverse cognition in IIH. This result may suggest that adiposity driven inflammation is not an important contributor to cognitive dysfunction in IIH.

### Reduction in intracranial pressure improves deficits in sustained attention

The role of intracranial pressure in cognitive impairment in IIH has not yet been fully established.^20^ While some investigators have noted that attention, psychomotor speed and executive functions were correlated with lumbar puncture pressure,^15^^,^^17^ others have not.^20^ In our study, improvements in executive function over 12 months were correlated with changes in lumbar puncture opening pressure. Furthermore, we demonstrate that acute changes in intracranial pressure following lumbar puncture rapidly improve executive function. Rapid improvement in cognition has also been noted in another cerebrospinal fluid disorder, idiopathic normal pressure hydrocephalus following CSF drainage.^55–57^ The relevance of raised intracranial pressure impacting cognitive performance may also be relevant in other diseases of raised intracranial pressure, including space-associated neuro-ocular syndrome.^58^

### Multifactorial cognitive dysfunction in idiopathic intracranial hypertension

There are numerous factors potentially contributing to the cognitive dysfunction in IIH. We have identified a number of factors potentially contributing to IIH cognitive performance in addition to intracranial pressure. Headache severity at the time of testing was associated with diminished attentional control. The role of headache pain on cognition in other settings is not conclusive, with conjunction as to whether it is the pain itself that could impact cognition or the underlying disease state that drives the headache pain.^59^^,^^60^ Here, there was no relationship between headache frequency or mean headache severity over the previous month, which suggests that it is the pain severity itself, at the time of testing, that directly impacts cognition. Depression and anxiety are highly prevalent in the IIH population.^2^^,^^48^^,^^61^^,^^62^ In other diseases, these comorbidities have been shown to impair memory, executive function, sustained attention and learning.^63–65^ We found an association between depression scores (HAD-D and SF-36 PCS, mental component summary score) and multiple cognitive domains. We postulate that identifying and treating anxiety and depression in IIH may improve cognitive function, as it does in other conditions.^66^

Obstructive sleep apnoea is a comorbidity found in IIH.^67^ Obstructive sleep apnoea is known to impact cognitive function (attention, working memory, episodic memory and executive functions).^27^^,^^49^^,^^68^ Our data demonstrated that in IIH, obstructive sleep apnoea indices (apnoea–hypopnea index, lowest oxygen desaturation and overnight desaturation index) were associated with impaired working memory. We also demonstrated that following 12 months of a weight loss intervention, reduction in obstructive sleep apnoea measurements (apnoea–hypopnea index and overnight desaturation index) was associated with improvement in executive function. We suggest that treating obstructive sleep apnoea in IIH with continuous positive airway pressure could modify cognitive dysfunction.^69^

### Serum cortisol and cognition in idiopathic intracranial hypertension

We have shown that working memory was associated with serum cortisol levels in IIH. Working memory was not identified as an impaired domain in IIH but this was in comparison to a control cohort with obesity. Obesity is an established driver for altered cortisol metabolism.^70^ Intracellular glucocorticoid levels are regulated by 11β HSD1 through conversion of inactive cortisone to active cortisol.^9^^,^^70^ IIH has been previously characterized as demonstrating a profile of increased global 11β HSD 1 activity, with levels falling in line with improving intracranial pressure.^9^ Therapeutic inhibition of 11β HSD 1 activity in IIH has been shown to improve intracranial pressure and markers of systemic metabolism.^10^^,^^11^ Importantly, inhibition of 11β HSD 1 activity improves memory in rodents,^71^^,^^72^ and inhibitors are under investigation to improve cognitive function in other settings. The effects of 11β HSD 1 inhibition on cognition in IIH have not been assessed but would be of future interest.

### Impaired attention in idiopathic intracranial hypertension impacts ability to perform reliable visual fields

Our study has shown that impaired attention and executive function impacts the ability to reliably perform visual field assessments. We report a consistent association with markers of visual field test reliability.^14^ Visual field testing has traditionally been the key measure of visual function in patients with IIH, although difficulties in test reliability are well recognized in this disease. Trials have demonstrated that 21% of visual field tests were performed unreliably and discarded.^52^ Our data suggest that impaired ability to perform attention and sustained attention tasks may be a key contributing factor. The poor performance is unlikely to be driven by headache pain as there was no association between visual field parameters and headache severity at the time of testing. Our results are consistent with studies in other disease areas, which have noted that impaired attention at the time of visual field testing influences test performance.^73^^,^^74^ These results are clinically relevant and may call into question the appropriateness of reliance on visual field test to guide treatment decision in active IIH.

### Strengths

This is the largest prospective IIH cohort analysed in a cognitive study, further strengthened by its longitudinal design allowing follow-up after 12 months. The assessment at 12 months allowed meaningful evaluation of the role of weight loss and changes in intracranial pressure. Our study also featured a BMI and intelligence matched control cohort which controlled for the confounding effects of obesity on cognition. The interventional aspect of the study allowed the comparison of the acute effect of decreasing intracranial pressure via lumbar puncture in both IIH and control participants.

### Limitations

There are a number of potential confounds that could have influenced the analysis of the IIH cohort compared to controls. The study was limited by a smaller number of controls than cases of IIH. While regression models could have been used to account for differences in gender and BMI in the control cohort, matching was felt to be a superior approach, although this did limit the number of control subjects eligible for inclusion. Additionally, control subjects underwent lumbar puncture, an invasive test, the number of which was limited due to ethical considerations. While the age of the IIH patients was significantly younger than controls, we feel this is unlikely to account for significant differences in executive function in this age group.^75–77^ We were not able to match for drug use between IIH and control participants. Topiramate was used by only 9% of IIH patients (Supplementary Table 1) and is known to impact on cognition.^78^ Another drug used in IIH is acetazolamide, and this is not known to impact cognitive function.^18^ This was the largest detailed study of cognition in IIH and the prolonged follow-up at 12 months was noteworthy compared to previous trials; however, this did lead to missing data over time which decreased the amount of analysable data. As participants had been exposed to cognitive tests at baseline, it is important to consider a potential learning effect to cognitive results at 12 months; however, we mitigated for this by using variations in the bespoke cognitive testing paradigms.

## Conclusion

In this prospective, longitudinal case-controlled study, we have shown that executive function is impaired in IIH. We have demonstrated reversibility of cognitive impairment with improvement in executive function, in line with decreased intracranial pressure. The cognitive impairment in IIH is multifactorial, being influenced by headache severity at the time of testing, depression and obstructive sleep apnoea. Impaired cognition is not widely appreciated in IIH by treating physicians, although is often articulated by patients. Our study improves the understanding of this complex multifactorial morbidity and suggests that cognition in IIH could be improved through reducing intracranial pressure, as well as managing depression, obstructive sleep apnoea and headache. Importantly, we highlight that IIH patients are likely to have inherent difficulties reliably performing visual field testing due to impaired attentional control.

## Supplementary material

Supplementary material is available at *Brain Communications* online.

## Supplementary Material

fcab202_Supplementary_Data

## Abbreviations

11β HSD 1 =: 11β hydroxysteroid dehydrogenase type 1
BMI =: body mass index
HAD-A =: hospital anxiety score
HAD-D =: hospital depression score
HIT-6 =: headache impact test-6
IIH =: idiopathic intracranial hypertension
PCS =: physical component summary score
SD =: standard deviation
SF =: short form
SITA =: Swedish Interactive Thresholding Algorithm
