# Supplementary material for: Cognitive performance in idiopathic intracranial hypertension and relevance of intracranial pressure
Source: Brain Commun. 2021 Sep 2;3(3):fcab202. doi: 10.1093/braincomms/fcab202 (PMC8421706; doi:10.1093/braincomms/fcab202)
Supplement: fcab202_Supplementary_Data [file fcab202_supplementary_data.docx]

**Supplementary Material**

**Cognitive performance in Idiopathic Intracranial Hypertension and relevance of intracranial pressure**

Olivia Grech MRes^1,2,^*, Andrew Clouter PhD^3^*, James L Mitchell MRCP^1,2,4^*, Zerin Alimajstorovic PhD^1,2^, Ryan S Ottridge MPhil^5^, Andreas Yiangou MRCP^1,4^, Marianne Roque MD DPBO^6^, Abd A Tahrani PhD^2,7,8^, Matthew Nicholls^2,7^, Angela E Taylor PhD^2,7^, Fozia Shaheen PhD^2,7^, Wiebke Arlt MD DSc^2,7,9^, Gareth G Lavery PhD^2,7^, Kimron Shapiro PhD^10^, Susan P Mollan FRCOphth^6^, Alexandra J Sinclair PhD^1,2,4^.

*Each author contributed equally

**Affiliations**

1. Metabolic Neurology, Institute of Metabolism and Systems Research, University of Birmingham, Edgbaston, B15 2TT, UK.
2. Centre for Endocrinology, Diabetes and Metabolism, Birmingham Health Partners, Birmingham, B15 2TH, UK.
3. Department of Psychology, Nottingham Trent University, Nottingham, NG1 5LT, UK.
4. Department of Neurology, University Hospitals Birmingham NHS Foundation Trust, B15 2TH, UK.
5. Birmingham Clinical Trials Unit, College of Medical and Dental Sciences, University of Birmingham, B15 2TT, UK.
6. Birmingham Neuro-Ophthalmology Unit, University Hospitals Birmingham NHS Foundation Trust, B15 2TH, UK.
7. Institute of Metabolism and Systems Research, University of Birmingham, Birmingham, B15 2TT, UK.
8. Department of Endocrinology and Diabetes, University Hospitals Birmingham NHS Foundation Trust, B15 2TH, UK
9. National Institute for Health Research (NIHR), Birmingham Biomedical Research Centre, University of Birmingham and University Hospitals Birmingham NHS Foundation Trust, Birmingham, B15 3GW, UK.
10. Centre for Human Brain Health, School of Psychology, University of Birmingham, Edgbaston, B15 2TT, UK.

**Supplementary methods**

**Participants**

IIH:WT was a five year randomized, controlled, parallel-group, multicenter trial. IIH:WT recruited participants at three UK National Health Service (NHS) hospitals between July 25, 2014 and May 25, 2017. Participants were identified from neurology and ophthalmology clinics from seven NHS hospitals. The National Research Ethics Committee West Midlands approved the trial (14/WM/0011).

Exclusion criteria were: pregnancy, significant comorbidity (including Cushing’s, Addison’s or use of steroids), undergone optic nerve sheath fenestration (due to their effects on long term OCT outcomes), specific medical or psychiatric contraindication for bariatric surgery and inability to provide informed consent. Those with central visual field defects that would impair ability to perform the screen based cognitive assessments were excluded. Those with a previous CSF shunt were included, but only if the shunt had failed and they had recurrence of active papilloedema (and ICP >25cmCSF), thus everyone in the cohort had active disease. For controls inclusion criteria were: female, BMI >35kg/m^2^, able to give informed consent and aged between 18 and 55 years. Exclusion criteria were: pregnancy, inability to give informed consent and diagnosis of IIH.

**Clinical measurements**

**Steroid hormone profiling**

After an internal standard mixture was added to 400 µl of serum, steroids were extracted via liquid/liquid extraction with 2 ml of tert-butyl methyl ether (MTBE). The MTBE layer was removed, evaporated to dryness, and reconstituted in methanol/water prior to LC-MS/MS analysis. The extracts were analyzed on a Xevo TQ-XS triple quadrupole mass spectrometer (Waters) coupled to an Acquity ultra-high performance liquid chromatography system (UPLC) (Waters). Steroids were separated on a HSS T3, (1.8 µm) column (Waters) using a methanol/water gradient (both with 0.1 % formic acid). Starting conditions was 45% methanol, which was held for 1 minute, followed by a linear gradient to 75 % methanol at 5 minutes. Subsequently, the column was washed at 98 % methanol and reconditioned at starting condition prior to the next injection. Steroid hormones were identified and quantified via comparison to reference standards; positive identification was confirmed via matching retention time and two identical mass transitions. Steroid hormones quantified were cortisol and cortisone. The calibration series ranged from 0.01 to 250 ng/ml (including a blank and a 0 ng/ml calibrator).

**Cognitive tests**

**Raven’s Standard Progressive Matrices (fluid intelligence)**

The Raven’s Standard Progressive Matrices fluid intelligence test was given in accordance with the standard instructions. The test consists of five sections, with 12 problems in each section. The problems get progressively more difficult within each section. Participants complete the task using a scoring sheet to mark their responses. The measurements for analysis are the total percentage correct and the time taken to complete the test.

**Attention network test**

The attention network test is used to measure the alerting, orienting, and executive components of attention, and the interactions among the three attention networks.^1-3^ Each trial was 3.5 seconds long, consisting of a fixation cross in the middle of the screen throughout the trial. After 1 second, on half of trials, an orienting cue appeared above or below the central fixation cross for 0.4 seconds. If the orienting cue had not appeared at 1 second, it would appear above or below fixation for 0.4 seconds at 1.4 seconds. Also at 1.4 seconds, on half of trials, an auditory alerting tone was presented for 0.1 seconds. At 1.8 seconds, five arrows appeared either above or below the central fixation cross for 1.7 seconds or until response. The target display was composed of a target central arrow pointing towards the left or right, and two flanking arrows on either side of the central arrow, all of which were pointing in the same direction (left or right). The task was to press the left or right arrow key on the keyboard to indicate whether the central arrow was pointing to the left or the right, as quickly and accurately as possible. If the target display appeared in the same location as the orienting cue, the orienting cue was valid; otherwise the orienting cue was invalid. If the flanking arrows were pointing in the same direction as the central target arrow, they were congruent with the target, if they were pointing in the opposite direction they were incongruent.

Participants completed a practice block of 24 trials before completing two blocks of 128 trials. The task has a two (auditory signal: yes or no) x two (orienting cue: valid or invalid) x two (flanker congruency: congruent or incongruent) design, such that there were 16 trials per condition in each block (32 trials per condition in total). The trials were presented in a random order in each block. Measurements for analysis included reaction times for correct responses and percentage correct. Condition differences (the alerting effect, the orienting effect, and the flanker effect) were computed as the difference in average reaction times and percentage of correct responses in each condition comparison (no alerting - alerting; invalid orienting cue - valid orienting cue; incongruent flankers - congruent flankers).

**Operation span task**

The operation span verbal working memory task (^4^ modified from ^5^) required that participants try to remember a series of words in the correct serial order, while trying to solve mathematical problems. Each trial started with the presentation of a math problem in the general form of “is (a x b) +/- c = d?”. On each trial, participants read the equation out loud followed by “yes” or “no”, if the equation was correct or incorrect. Half of presented equations were correct. Immediately following the response to the equation, a word appeared on the screen for one second, which participants read aloud. Following a 0.5 second delay, another trial was presented, or the recall instruction was presented. When presented with the recall instruction, participants recalled, out loud, the words that were presented in that block of trials, in the serial order in which they were presented. If a word could not be recalled, participants were instructed to replace the word with “can’t remember” to preserve the serial order of words. Blocks consisted of two, three, four or five trials before the recall instruction, with three blocks of each size presented in a pseudorandom order. The measurement used for analysis was the overall percentage correct of 36 trials (blocks of trials with a size of two were excluded from the analysis due to performance ceiling effects).

**Sustained attention task**

In the sustained attention task, 225 digits (25 of each of the digits from 1-9) were presented for 0.25 seconds each. They were immediately followed by a mask (comprised of the overlaid capital letters O and X) for 0.9 seconds, to ensure speeded evaluation. The digits and mask on a given trial were of varying size on the screen (five sizes for each digit: 2, 2.5, 3, 3.5, and 4 normalised units, which were presented five times each). Participants were given the target number six, and only pressed the response button when the target number appeared (25 times total) and were told to respond as quickly and as accurately as possible. The task began after a practice block of 18 trials. Measurements for analysis included reaction time for correct responses, total percentage correct, and percentage correct to the target (errors of omission: when the target was presented but the response was incorrectly withheld).

**Word span task**

The word span task is the verbal short-term memory counterpart to the operation span task. On each trial, the participant is presented with a word on the computer screen for 1 second, followed by a 0.5 second delay, and the presentation of another word or the recall instruction. Participants read each word out loud when it appeared. When presented with the recall instruction, participants recalled aloud, the words presented during that block of trials in the correct serial order. As with the operation span task, participants were instructed to replace forgotten words with ‘can’t remember’ to preserve the serial order of the recalled words. Blocks consisted of 2, 3, 4, 5, 6, or 7 words before the recall instruction, with each size presented three times in a pseudorandom order. The measurement used for analysis was the overall percentage correct of 66 trials (blocks of trials with sizes 2 and 3 were excluded from the analysis due to ceiling effects).

**Sustained attention to response task**

The sustained attention to response task was presented in exactly the same fashion as the sustained attention task, except that the instructions were for the participant to press the response key for every digit that appeared except if the target (the digit three) appeared.^6, 7^ The task began after a practice block of 18 trials. Measurements for analysis included reaction time for correct responses, total correct, total correct to the target (errors of commission: when the target was presented but a response was incorrectly made). The sustained attention to response task was carried out twice in a sub-group of IIH and control participants before and after a lumbar puncture. This was to allow the assessment of the effect of acute reduction in intracranial pressure.

**Supplementary References**

1. Fan J, McCandliss BD, Sommer T, Raz A, Posner MI. Testing the efficiency and independence of attentional networks. *J Cogn Neurosci*. Apr 1 2002;14(3):340-7. doi:10.1162/089892902317361886

2. Callejas A, Lupiàñez J, Funes MJ, Tudela P. Modulations among the alerting, orienting and executive control networks. *Exp Brain Res*. Nov 2005;167(1):27-37. doi:10.1007/s00221-005-2365-z

3. Ishigami Y, Klein RM. Repeated measurement of the components of attention using two versions of the Attention Network Test (ANT): stability, isolability, robustness, and reliability. *J Neurosci Methods*. Jun 30 2010;190(1):117-28. doi:10.1016/j.jneumeth.2010.04.019

4. Engle RW, Tuholski SW, Laughlin JE, Conway ARA. Working memory, short-term memory, and general fluid intelligence: a latent-variable approach. *J Exp Psychol Gen*. Sep 1999;128(3):309-331. doi:10.1037//0096-3445.128.3.309

5. Kane MJ, Hambrick DZ, Tuholski SW, Wilhelm O, Payne TW, Engle RW. The Generality of Working Memory Capacity: A Latent-Variable Approach to Verbal and Visuospatial Memory Span and Reasoning. *Journal of Experimental Psychology: General*. 2004;133(2):189-217. doi:10.1037/0096-3445.133.2.189

6. Robertson IH, Manly T, Andrade J, Baddeley BT, Yiend J. `Oops!': Performance correlates of everyday attentional failures in traumatic brain injured and normal subjects. *Neuropsychologia*. 1997/05/19/ 1997;35(6):747-758. doi:<https://doi.org/10.1016/S0028-3932(97)00015-8>

7. Manly T, Robertson IH, Galloway M, Hawkins K. The absent mind: further investigations of sustained attention to response. *Neuropsychologia*. Jun 1999;37(6):661-70. doi:10.1016/s0028-3932(98)00127-4

**Supplementary Figure**

**
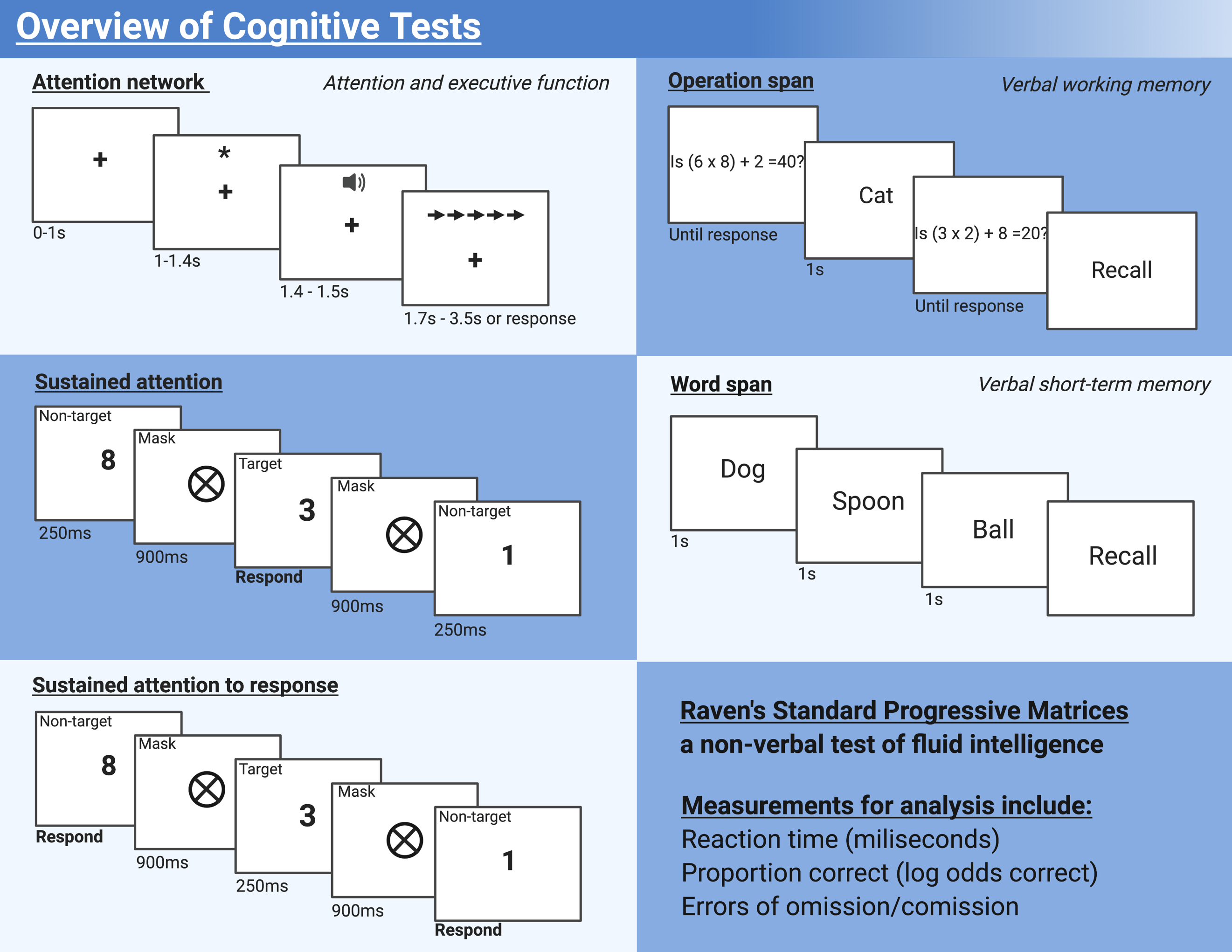
**

**Supplementary Fig. 1 Overview of cognitive tests completed by control and IIH participants.**

All participants completed the tasks in order: the attention network test, operation span, sustained attention, word span, and the sustained attention to response task. **Attention network:** A fixation cross would remain in the middle of the screen throughout the 3.5s trial. In half of the trials; an orientating cue appeared above or below, after that an alerting tone was presented and if the cue had not appeared previously it did at this time. Then five arrows would appear below or above the fixation cross. The task was to press the left or right arrow key to indicate if the central arrow was pointing in the left or right direction. **Operation span:** Participants were required to remember a series of words in the correct order while trying to solve mathematical problems. The participants were required to read the equations aloud followed by yes or no and asked to recall words at the end of the trial. **Sustained attention**: Participants were given the target number 6 and presented with 225 digits (25 of each of the digits 1-9) which were masked between digits. Participants were required to respond when the target number appeared. **Word span**: Participants are presented with a series of words and asked to recall the list in order. **Sustained attention to response**: Presented in the same fashion as SA task however participants were required to press the response key for every digit that appeared except the target number (3).

Test results were measured in reaction time (milliseconds), proportion correct (log odds correct) and errors of omission/commission (percentage correct when the target was presented but the response was incorrectly withheld).

**Supplementary Table Guide**

**Supplementary Table 1.** Summary of current medication use in IIH participants at baseline.

**Supplementary Table 2.** Descriptive statistics for attention network test conditions in IIH and controls.

**Supplementary** **Table 3.** Descriptive characteristics of IIH participants at baseline and 12 months follow up

**Supplementary Table 4.** Baseline and follow up descriptive statistics and within-group comparisons

**Supplementary Table 5.** Comparison of change in cognitive performance between community weight management intervention and surgery groups over 12 month period

**Supplementary Table 1.** Summary of current medication use in IIH participants at baseline.

| **Medication** | **IIH**  **(n =66)** | **Control**  **(n = 25)** |
| --- | --- | --- |
| **Acetazolamide, n (%)** | 19 (29%) | 0 |
| Daily dose (mg); mean (SD) | 855 (509) | - |
| **Topiramate, n (%)** | 6 (9%) | 1 (4%) |
| Daily dose (mg); mean (SD) | 75 (67) | 200 |
| **Diuretics** | 3 (5%) | 0 |
| Bendroflumethiazide | 1 (33%) | - |
| Furosemide | 1 (33%) | - |
| Co-amilofruse | 1 (33%) | - |
| **Antihypertensive** | 5 (8%) | 2 (8%) |
| B-blockers | 1 (20%) | 0 |
| ACE inhibitors | 1 (20%) | 2 (100%) |
| A-II antagonist (ARB) | 1 (20%) | 0 |
| Other | 2 (40%) | 0 |
| **Other headache preventatives** | 18 (27%) | 2 (8%) |
| Beta-blocker | 1 (6%) | 2 (100%) |
| Tricyclic | 7 (39%) | 0 |
| Anticonvulsant | 8 (44%) | 0 |
| Other | 2 (11%) | 0 |

ACE = Angiotensin-converting-enzyme; ARB = angiotensin II receptor blockers

|  |  | **Control** | **IIH** |
| --- | --- | --- | --- |
| **Condition** | **Measure** | Mean (SD), n | Mean (SD), n |
| Alerting | RT | 0.693 (0.145), 19 | 0.696 (0.090), 53 |
| No Alerting | RT | 0.711 (0.153), 19 | 0.713 (0.092), 53 |
| Alerting | Correct | 0.983 (0.028), 19 | 0.960 (0.059), 53 |
| No Alerting | Correct | 0.975 (0.048), 19 | 0.961 (0.061), 53 |
| Valid Orienting | RT | 0.667 (0.156), 19 | 0.663 (0.092), 53 |
| Invalid Orienting | RT | 0.737 (0.145), 19 | 0.746 (0.091), 53 |
| Valid Orienting | Correct | 0.981 (0.039), 19 | 0.967 (0.055), 53 |
| Invalid Orienting | Correct | 0.977 (0.039), 19 | 0.954 (0.066), 53 |
| Congruent Flankers | RT | 0.640 (0.152), 19 | 0.642(0.088), 53 |
| Incongruent Flankers | RT | 0.764 (0.150), 19 | 0.767(0.097), 53 |
| Congruent Flankers | Correct | 0.997 (0.006), 19 | 0.985 (0.031), 53 |
| Incongruent Flankers | Correct | 0.961 (0.068), 19 | 0.936 (0.1020, 53 |

**Supplementary Table 2.** Descriptive statistics for attention network test conditions in IIH and controls.

RT = Reaction time; Correct = proportion correct

**Supplementary** **Table 3.** Descriptive characteristics of IIH participants at baseline and 12 months follow up

| **Clinical measurement** | **Baseline** | **12 months** | **p** |
| --- | --- | --- | --- |
|  | **Mean (SD), n** | **Mean (SD), n** |  |
| Body Mass Index (BMI), kg/m^2^ | 43.9 (7.0), 66 | 39.0 (8.8), 59 | <0.001 |
| Intracranial opening pressure (cmCSF) | 34.7 (5.7), 66 | 29.0 (7.7), 54 | <0.001 * |
| Intracranial closing pressure (cmCSF) | 19.4 (3.8), 61 | 17.7 (4.6), 52 | 0.062 |
| Headache severity day of test | 3.5 (2.8), 61 | 2.1 (3.0),51 | 0.009 * |
| Monthly headache days | 22.2 (8.0), 63 | 14.8 (11.6), 53 | <0.001 * |
| Headache severity | 5.0 (2.0), 63 | 3.6 (2.9), 53 | <0.001 * |
| Headache disability (HIT-6) | 64.7 (7.3), 65 | 58.5 (10.7), 55 | <0.001 * |
| Serum IL-6 | 6.0 (2.5), 61 | 5.7 (2.4), 50 | 0.313 |
| CSF IL-6 | 6.5 (15.8), 54 | 3.6 (1.9), 47 | 0.269 |
| OCT RNFL thickness (µM) | 139.6 (5.8), 59 | 106.3 (28.0), 57 | <0.001 * |
| Hospital anxiety and depression scale – Anxiety score (HAD A) | 10.3 (4.9), 65 | 9.9 (4.9), 57 | 0.466 |
| Hospital anxiety and depression scale - Depression score (HAD D) | 7.6 (4.5), 65 | 6.70 (4.7), 57 | 0.108 |
| Quality of life (PCS) | 28.7 (12.7), 60 | 37.7 (14.9), 53 | <0.001 * |
| Quality of life (MCS) | 37.7 (11.0), 60 | 38.9 (12.2), 53 | 0.633 |
| Apnea-hypopnea index | 14.1 (20.5), 40 | 12.4 (21.0), 20 | 0.025 * |
| Humphrey visual field mean deviation | -3.6 (3.7), 65 | -2.4 (2.5), 58 | <0.001 * |

HIT-6 = headache impact test; IL-6 = interleukin-6; MCS = mental component score; OCT = optical coherence tomography; PCS = physical component score.

|  |  |  |  | **Baseline** | **Follow up** |  |  |
| --- | --- | --- | --- | --- | --- | --- | --- |
| **Cognitive Test** | **Measure** | **Group** | ***n*** | **Score (SD)** | **Score (SD)** | **Change** | **p** |
| **Attention network (averaged)** | RT | CWI | 18 | 690 (89) | 653 (83) | -37 | 0.079 |
|  |  | Surgery | 21 | 699 (78) | 661 (99) | -39 | 0.032 |
|  | Correct | CWI | 18 | 0.971 (0.041) | 0.985 (0.021) | 0.013 | <0.001 |
|  |  | Surgery | 21 | 0.957 (0.076) | 0.950 (0.108) | -0.007 | 0.036 |
| **Sustained attention** | RT | CWI | 18 | 464 (49) | 436 (45) | -28 | 0.001 |
|  |  | Surgery | 16 | 479 (55) | 459 (40) | -0.020 | 0.087 |
|  | Correct | CWI | 18 | 0.996 (0.005) | 0.995 (0.007) | -0.001 | 0.516 |
|  |  | Surgery | 16 | 0.996 (0.004) | 0.994 (0.009) | -0.002 | 0.206 |
|  | Target Correct | CWI | 18 | 0.987 (0.023) | 0.977 (0.039) | -0.010 | 0.239 |
|  |  | Surgery | 16 | 0.976 (0.027) | 0.962 (0.059) | -0.014 | 0.190 |
| **Sustained attention to response** | RT | CWI | 20 | 400 (79) | 357 (61) | -0.043 | 0.012 |
|  |  | Surgery | 16 | 380 (66) | 350 (59) | -0.029 | 0.097 |
|  | Correct | CWI | 20 | 0.910 (0.082) | 0.892 (0.113) | -0.017 | 0.007 |
|  |  | Surgery | 16 | 0.894 (0.041) | 0.901 (0.067) | 0.008 | 0.294 |
|  | Target Correct | CWI | 20 | 0.716 (0.296) | 0.679 (0.323) | -0.037 | 0.007 |
|  |  | Surgery | 16 | 0.562 (0.247) | 0.628 (0.310) | 0.066 | 0.300 |
| **Word span** | Correct | CWI | 21 | 0.647 (0.114) | 0.630 (0.116) | -0.017 | 0.337 |
|  |  | Surgery | 21 | 0.594 (0.182) | 0.660 (0.148) | 0.066 | <0.001 |
| **Operation span** | Correct | CWI | 20 | 0.547 (0.201) | 0.639 (0.193) | 0.092 | 0.181 |
|  |  | Surgery | 19 | 0.631 (0.180) | 0.662 (0.143) | 0.030 | 0.378 |

**Supplementary Table 4.** Baseline and follow up descriptive statistics and within-group comparisons

CWI = Community weight management intervention; RT = Reaction time; Correct = proportion correct; Target correct = proportion correct on target-present trials; Scores expressed as mean (SD) and compared using paired t-tests or z-tests as appropriate.

**Supplementary Table 5.** Comparison of change in cognitive performance between community weight management intervention and surgery groups over 12 month period

| **Interaction** | **Measure** | **p** |
| --- | --- | --- |
| Attention network task alerting (yes – no) x Group x Session | RT | 0.701 |
|  | Correct | 0.124 |
| Attention network task alerting (valid – invalid) x Group x Session | RT | 0.581 |
|  | Correct | 0.217 |
| Attention network task flanker (incongruent – congruent) x Group x Session | RT | 0.037 |
|  | Correct | 0.800 |
| Sustained attention x Group x Session | RT | 0.541 |
|  | Correct | 0.741 |
|  | Target Correct | 0.911 |
| Sustained attention to response x Group x Session | RT | 0.569 |
|  | Correct | 0.009 |
|  | Target Correct | 0.011 |
| Word span x Group x Session | Correct | 0.001 |
| Operation span x Group x Session | Correct | 0.420 |
|  |  |  |

RT = Reaction time (seconds); Correct = proportion correct; Target correct = proportion correct on target-present trials; comparisons made using mixed (within- and between-groups) analysis of variance.
